# Study on the characteristics of increased mechanical stiffness according to changes in LCP shape to reinforce clavicle fractures

**DOI:** 10.1038/s41598-024-56588-z

**Published:** 2024-03-16

**Authors:** Soo Min Kim, Seong-tak Kim, Dong-woon Han, Dae-Geun Kim

**Affiliations:** 1 ICT Convergence Research Division, Realistic Media Research Center, Gumi Electronics and Information Technology Research Institute (GERI), Gumi, 39253 Republic of Korea; 2 Functional Materials and Components R &D Group, Korea Institute of Industrial Technology (KITECH), Wonju, 26336 Republic of Korea; 3 Department of Orthopedic Surgery, College of Medicine, Soonchunhyang University Gumi Hospital, Gumi, 39371 Republic of Korea

**Keywords:** Clavicle, Locking screw plate, Double-shaped partial wing structure, Medical research, Engineering

## Abstract

The clavicle has various anatomic shapes unique to each individual. Additionally, with the increase in high-energy traumas such as sports injuries and traffic accidents, the patterns of fractures become complex and complicated. Thus, there is a need for a variety of shapes of locking compression plates (LCP) to accommodate different types of fractures and facilitate quicker rehabilitation. The aim of this study is to present different types of LCP that secure fracture fragments and distribute stress evenly, in comparison to typical anatomical LCPs, for reinforcing clavicle fractures. Three models were compared in this study: the typical shape, the center hole removed shape, and the double-curved wing shape. The DICOM (Digital Imaging and Communications in Medicine) file obtained from the computed tomography scan of the patient’s clavicle was used to extract the three-dimensional (3D) clavicle structure. Finite element analysis (FEA) simulation was employed to analyze the structural changes of the LCP under external forces. A reinforced jig was used to apply the same type of external force to each LCP, and an experiment was conducted to analyze the mechanical impact of the LCP’s structural characteristics. When comparing the stress values at the fracture zone point, resulting from the FEA simulation with applied bending forces, it was calculated that the stress dispersion effect was approximately ten times greater when transitioning from a typical LCP shape to a double-curved partial wing structure. Moreover, the ultimate stress increased 3.33 times, from 241.322 to 804.057 N, as the LCP design changed under cantilever bending conditions. This double-curved wing LCP design reduces stress concentration at the fracture site and minimizes stress in the fracture area when subjected to cantilever bending forces. Consequently, this newly designed LCP has the potential to decrease complications related to the plate and accelerate rehabilitation protocols.

## Introduction

Clavicle midshaft fractures (CMFs) are common fractures, accounting for approximately 44% of the scapular fractures^1–3^. In the past, both non-displaced and substantially displaced CMFs were usually managed conservatively and showed favorable outcomes^4^. However, with the increasing prevalence of high-energy injuries such as sports injuries and traffic accidents, CMFs are recently more often accompanied by displacement or comminution^3^. As a result, surgical intervention has become the preferred treatment for displaced or comminuted CMFs due to its higher rate of bone union and lower complication rate^5–8^.

When fixing a plate to treat CMFs, the cantilever bending force has the greatest impact compared to axial compression and rotational forces^9^. We have developed a locking screw cap designed to be inserted into the empty screw hole at the fracture site^9^. However, since clavicle anatomical shapes and fracture patterns can vary, not all metal plates may be suitable. Therefore, a locking screw cap alone is insufficient. Previous finite element analysis (FEA) studies have shown that increasing the thickness or width at the center of metallic plates reduces maximum stress and deformation^10^. Considering that the fracture site is typically located in the center of the plate, we have designed a plate with small wings on both sides, widening only the width at the center.

We hypothesized that the plate with small wings would exhibit greater strength than conventional plates under cantilever bending forces. We examined the stress on both the conventional locking plate and the plate with small wings, considering three forces: cantilever bending, axial compression, and rotational forces. Additionally, we used three-dimensional (3D) printers to create CMF models, conventional locking plates, and novel plates, which were then verified.

## Methods

### CT image preparation & 3D structure construction

The 3D clavicle model was created using axial images with a slice thickness of 1.0 mm from a normal left clavicle obtained through a computerized tomography (CT) scan. The images were taken from a 55-year-old female volunteer who provided written informed consent for the publication of clinical details and images.

**Figure 1:**
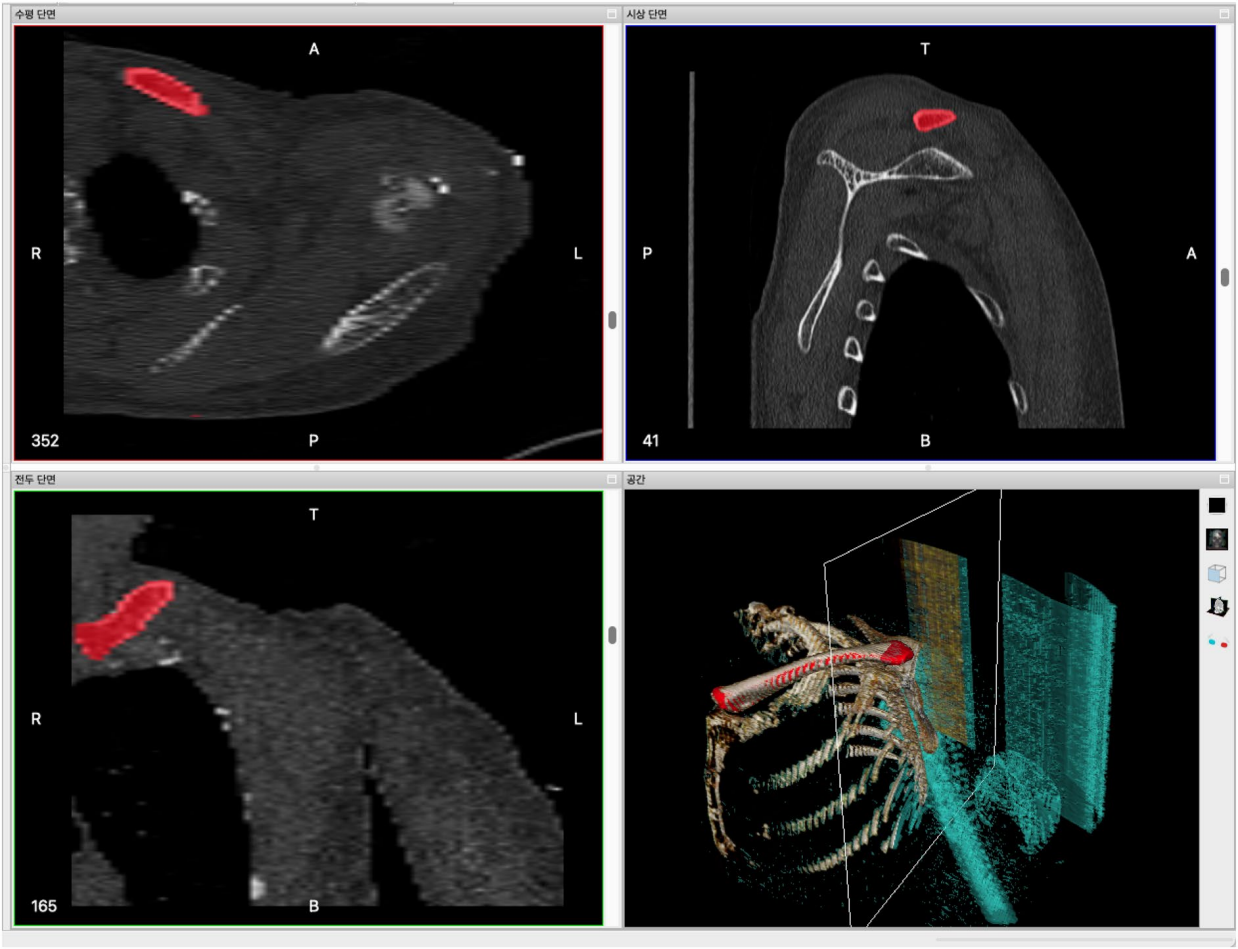
Image of CT(computerized tomography) cross-sectional photos synthesized and converted into a 3D structure using the InVesalius program.

The image file format from the CT scan of the patient’s clavicle is DICOM (Digital Imaging and Communications in Medicine). We utilized InVesalius software (v3.1.1, Center for Information Technology Renato Archer, Campinas, Brazil) to read the image file and extract the 3D structure of the clavicle. The shape of the clavicle was defined in each plane, taking into account its anatomical characteristics in the frontal, lateral, and cross-sectional directions of the CT image (Fig. 1). Afterwards, the 3D structure and clavicle shape were anatomically extracted. During the clavicle extraction process, some step-like irregularities may arise on the surface due to the 1.0 mm gap in the source CT image. To address this, we used Autodesk Meshmixer program (version 3.5) to smooth the surface, resulting in a more refined 3D clavicle image. The final output was saved as a stereo-lithography (STL) file, which was subsequently used for the FEA simulation.

The fracture site was created using Solidworks software (Dassault Systemes SolidWorks Corp.), with a simple fracture pattern located in the middle of the clavicle. To fix the fracture fragment, designed locking compression plates (LCP) were used to closely match the actual shape of the clavicle. The design principles for the customized LCP were as follows:

1. The LCP should be fixed to the clavicle using three screws on each side of the fracture site.
2. The angle of the screws that penetrate the clavicle should be adjusted in three or more directions, ensuring that the cross-sectional area of the clavicle at the screw location is similar on both sides.
3. The spacing between the screws used in the clavicle should be maintained, with the fracture area being at the center.

**Figure 2:**
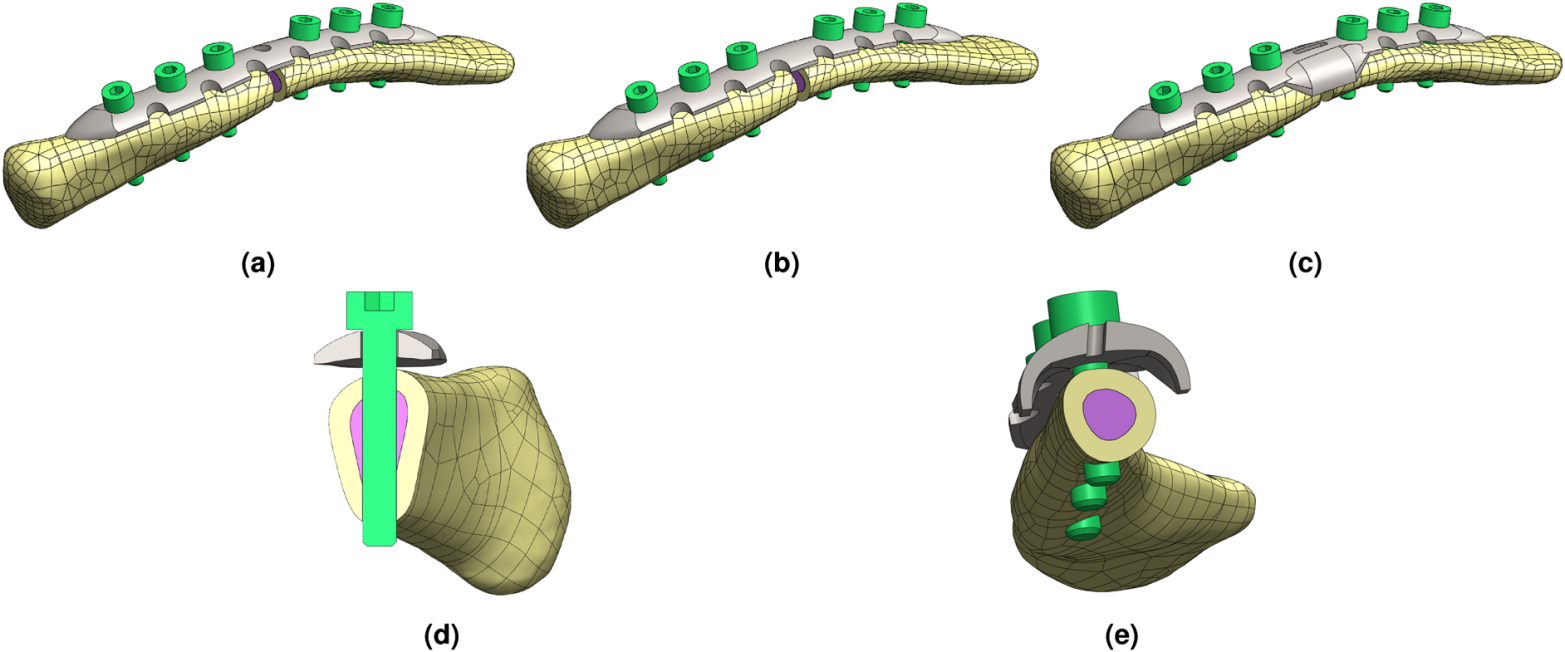
3D model of customized LCP design structures for clavicle and reinforcement with artificial fracture created using Solidworks, (**a**) typical shape customized design (Type 1), (**b**) berried-hole shape customized design (Type 2), (**c**) double-curved wing shape customized design (Type 3), (**d**) cross-section area of penetration bolt at M$_{1}$ hole, (**e**) cross-section area of double-curved wing shape LCP at FZ site.

Three models were created for the study. The first model was the typical LCP shape tailored to the clavicle morphology (Type 1). The second model was a typical LCP, but with the middle hole filled (Type 2). Lastly, the third model had double-curved wings (Type 3) (Fig. 2).

### FEA simulation

**Figure 3:**
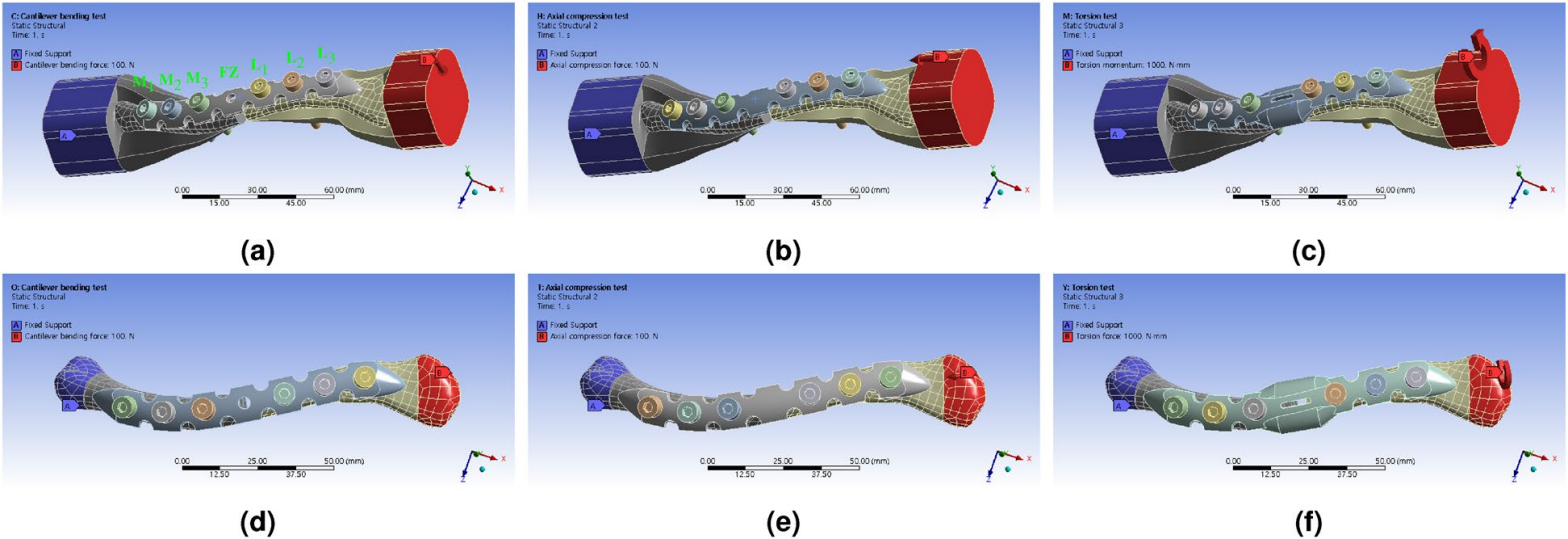
FEA simulation 3D model structure and boundary conditions for each area, combined with reinforced clavicle structure jig, (**a**) cantilever bending force applied to type 1 (Typical) LCP structure, (**b**) axial compression force applied to type 2 (Berried-hole) LCP structure, (**c**) torsion torque applied to type 3 (Double-curved wing) LCP structure, and combined with actual clavicle structure (**d**) cantilever bending force applied to type 1 (Typical) LCP structure, (**e**) axial compression force applied to type 2 (Berried-hole) LCP structure, (**f**) torsion torque applied to type 3 (Double-curved wing) LCP structure.

To accurately analyze the stress dispersion effect of the LCP and account for the complex dynamics of force transmission in the clavicle, we needed to address the variations in clavicle structure and minimize variables. For this purpose, we have redesigned the reinforcement model to prevent deformation of the structurally weak clavicle (Fig. 3). By fixing the LCP under the same conditions, the clavicle structure with reinforcement could be maintained so that it was equally exposed to the direction of the applied external force. This allowed for accurate analysis of the stress distribution effect of the LCP, as the transmitted external force could be concentrated entirely on the plate.

For the FEA simulation, the boundary conditions were set assuming that the surgery using the LCP has already reinforced the fractured clavicle structure. The ends of the clavicle where the fracture occurred were defined as either a fixation structure or a structure capable of applying force (Fig. 3). The left part of the clavicle was defined as the fixed support point, while the right part was identified as the region where the external force was applied. To analyze the stress distribution effect in the proposed LCP structure, the boundary conditions were set to apply cantilever bending force, axial compression force, and torsion torque.

The boundary conditions for the FEA simulation assumed a scenario where the clavicle fracture was fixed through surgery using the LCP. The analysis aimed to investigate the stress distribution effect in the proposed LCP structure by applying cantilever bending force, axial compression force, and torsion torque at the joint.

The mechanical properties of all materials^11–19^ we used in FEA simulations was showed (Table 1). To analyze the structure that can be seen in Fig. 3, a mesh was created under the condition of having mechanical physics preference. To create this mesh, the element order was set under program controlled conditions, and the resolution value was defined as 7. In addition, the Mesh Defeaturing function was activated, the transition condition was defined as Fast, and the Span Angle Center item was set to Corse. The Initial Size Seed condition for creating the mesh was defined as Assembly. The analysis results for the mesh created using the above boundary conditions are summarized in Table 2. The six simulation models conducted in this study have Element/Orthogonal quality and Skewness of Very Good grade or higher. During the simulation analysis process, no convergence difficulties or issues with physical descriptions occurred. To analyze the stress distribution effect of the LCP, calculations were conducted in static structure mode. As part of the analysis, seven points were considered for comparison: Medial (M) 1–3, fracture zone (FZ), and lateral (L) 1–3. These points were fixed, and the maximum equivalent stress, also known as the von-Mises stress ($\sigma _{m}$), was determined for each point.

**Table 1:** Mechanical properties of materials used in FEA simulation.

Mechanical properties	Maraging steel	Stainless steel	Titanium alloy	Cortical bone	Cancellous bone
Young’s modulus (MPa)	130,000	193,000	186,400	17,000	1,000
Bulk modulus (MPa)	108,330	169,300	155,330	14,167	833
Shear modulus (MPa)	50,000	73,664	71,692	6,539	385
Poisson’s ratio	0.3	0.31	0.3	0.3	0.3
Density (g/cm$^{3}$)	8.00	7.75	4.62	1.19	1.19

**Table 2:** Mesh information and quality analysis results used in FEA simulation based on LCP material and geometry type.

Clavicle materials	LCP type	Volume (mm$^3$)	Node	Element quality$^\text{a}$		Skewness$^\text{a}$		Orthogonal quality$^\text{a}$	
				Average	Deviation	Average	Deviation	Average	Deviation
Metal	1	85,796	1,035,418	0.85198	0.091198	0.2088	0.11848	0.78995	0.11701
	2	86,002	1,037,157	0.85162	0.091227	0.20939	0.11855	0.78934	0.11706
	3	86,390	1,043,721	0.85138	0.091574	0.20971	0.11888	0.78902	0.1174
Bone	1	19,744	303,229	0.81875	0.11004	0.25969	0.14253	0.73884	0.14076
	2	19,781	303,489	0.81891	0.11008	0.2596	0.14263	0.73892	0.14086
	3	20,169	310,256	0.81859	0.11085	0.25996	0.14336	0.73856	0.14159

$^\text{a}$Tetra 10 volume mesh.

The external force condition applied to the clavicle undergoing LCP treatment was assumed to be applied in the direction of the human body’s shoulder joint, and the direction of the human body’s medium was set as fixed. The types of applied forces defined in the analysis include cantilever bending force, axial compression force, and torsion, totaling three types. This form of external force is a simplification of the natural arm deflection, side support of the body with the arm, and arm rotation process based on the human shoulder joint. In this study, a cantilever bending force of 100 N, an axial compression force of 100 N, and a torsion of 1000 N$\cdot$mm were applied to the shoulder joint area^11,20–22^ (Fig. 3).

Furthermore, in order to distinguish the region where the max equivalent stress occurs, the stress distribution results inside the LCP area were extracted separately and set to be compared with the stress distribution results of the entire structure. Equivalent (von-Mises) strain values were extracted to confirm the magnitude of deformation under the same load conditions. The value of the strain rate can then be compared with the results of the stress-strain diagram in the subsequent verification test, as the load applied in the boundary condition remains the same.

### 3D printing process

**Figure 4:**
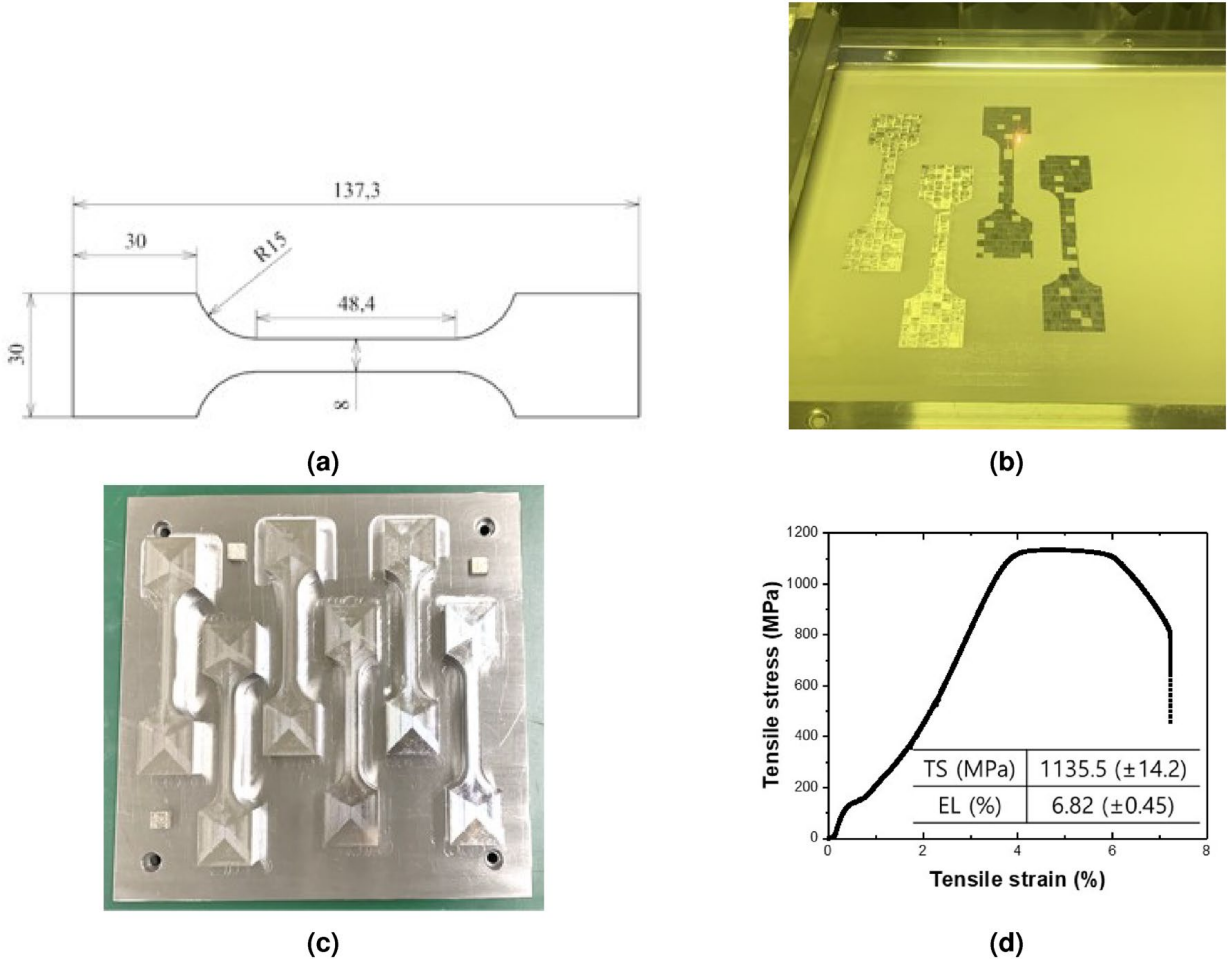
Information on 3D printing metal materials used in LCP and clavicle fabrication, (**a**) Tensile specimen, (**b**) 3D printing process of tensile specimen, (**c**) 3D printing product of tensile specimen, (**d**) Representative mechanical property of tensile specimens produced by 3D printing, The insert value shows the average value for 8 tensile specimens.

The LCP and bolts used have been made of titanium alloy. However, it was challenging to manufacture a customized structure using this material. To overcome this limitation, we employed a 3D printer that sintered metal powder with a laser to fabricate the customized structure. For this purpose, maraging steel was chosen as the metal powder material due to its similar tensile strength properties to titanium alloy. Specifically, 18Ni-300 maraging steel powder (Matsuura Machinery Co. Ltd., Japan) was utilized to manufacture the LCP and clavicle model. The composition of the powder is provided in Table 3, and its particle size distribution (measured using Malvern 2000 particle size distribution from Malvern Instrument) showed D10, D50, and D90 values of 22.8 $\upmu$m, 34.7 $\upmu$m, and 52.5 $\upmu$m, respectively. The build plate for additive manufacturing was made of S45C steel with a size of 245 $\times$ 245 $\times$ 20 mm. Additive manufacturing was performed using LUMEX Avance-25 (Matsuura Machinery Co. Ltd., Japan) using Yb-fiber laser. The process parameters used consist of a previously optimized conditions: laser power of 300 W, laser scanning speed of 700 mm/s, hatch spacing of 0.12 mm, and layer thickness of 0.05 mm. The Build plate temperature was performed at room temperature, and the working chamber was filled with nitrogen gas with 0.1 % of oxygen content. To compare the physical properties with titanium alloy, standard tensile test specimens were produced using 3D printing, following both ASTM and KS standards. These specimens exhibited similar tensile stress but lower tensile strain values compared to titanium alloys reported in the literature^23,24^ (Fig. 4).

**Table 3:** Composition.

Element	Ni	Co	Mo	Ti	Cr	Al	Si	Fe
wt%	17.8	8.8	4.9	0.7	0.2	0.1	0.1	Bal.

**Figure 5:**
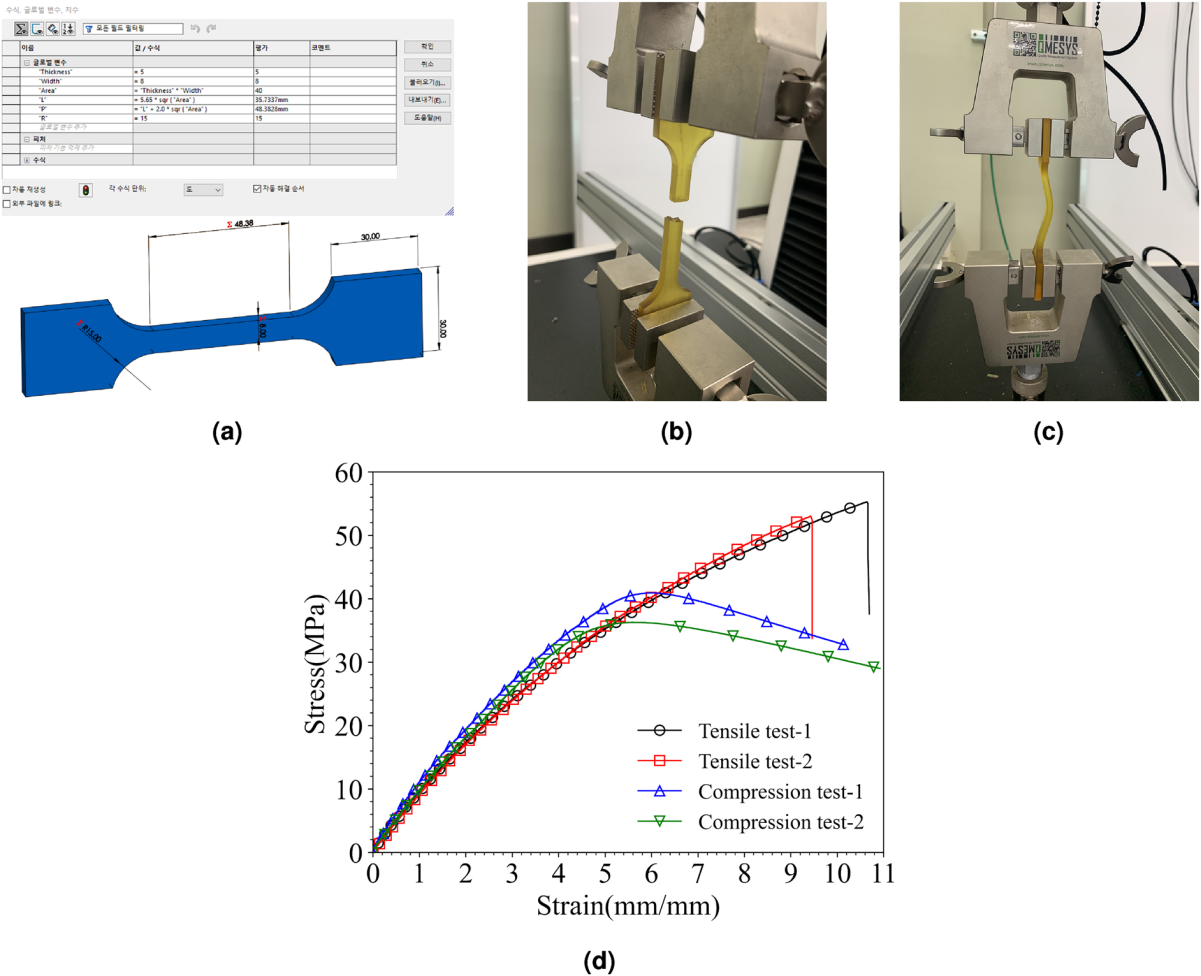
Tensile and compression testing process using UTM, (**a**) specimen design for standard tensile testing, (**b**) tensile test result, (**c**) compression test result, (**d**) stress-strain curve of tensile and compression test process.

For the formation of the clavicle model, Stereolithography (SLA) liquid crystal display ultraviolet resin 3D printers (ANYCUBIC PHOTON M3 MAX) were utilized to produce specimens that replicated the stiffness of an actual clavicle. The printing conditions were set with a layer thickness of 0.050 mm, a Z axis retract step speed of 1.5 mm/s, and a normal exposure time of 7 s. To ensure high hardness, bending strength, and tensile strength, a heat-resistant 3D printer resin material called HT100 was employed to create the 3D structure of the clavicle. The manufacturer (RESIONE) reports the mechanical properties of the cured HT100 resin as follows: flexural strength of 108 MPa, flexural modulus of 2.88 GPa, and tensile strength of 78 MPa^25^. To verify that the resin’s stiffness aligns with the specifications, a tensile test specimen was printed using a 3D printer, and a tensile test was conducted. The printed tensile specimen conformed to the KCTL-14B-plate type standard, with a designed thickness of 5 mm. The specimen had a defined width of 8 mm, resulting in a tensile test area of 40 mm$^{2}$. The gauge length of the specimen is 35.7337 mm, and the shoulder radius was 15 mm. The section without shoulders measured 48.3828 mm in length, and both the length and height of the grip section were set at 30 mm. By utilizing the aforementioned printing conditions, the output of the 3D resin printer took the form of a translucent yellow standard specimen (Fig. 5).

We conducted tensile and compression tests using a universal testing machine (UTM) (QMESYS QM100T model, South Korea). The tests were performed on printed standard specimens with a tensile speed of 0.01 mm/s. The stress-strain diagram obtained from both the tensile and compression tests is displayed in Fig. 5d. The ultimate tensile strengths were recorded as 55.27 MPa and 53.01 MPa, respectively, depending on the number of tests conducted. In the case of the compression test, the values recorded were 40.91 MPa and 36.28 MPa. These results reflect a deviation of approximately 30% when compared to the 78 MPa specified in the product specifications. However, it is worth noting that these measured values fall within the range of skeletal strength as reported in the references^26–30^.

### Mechanical property validation test

**Figure 6:**
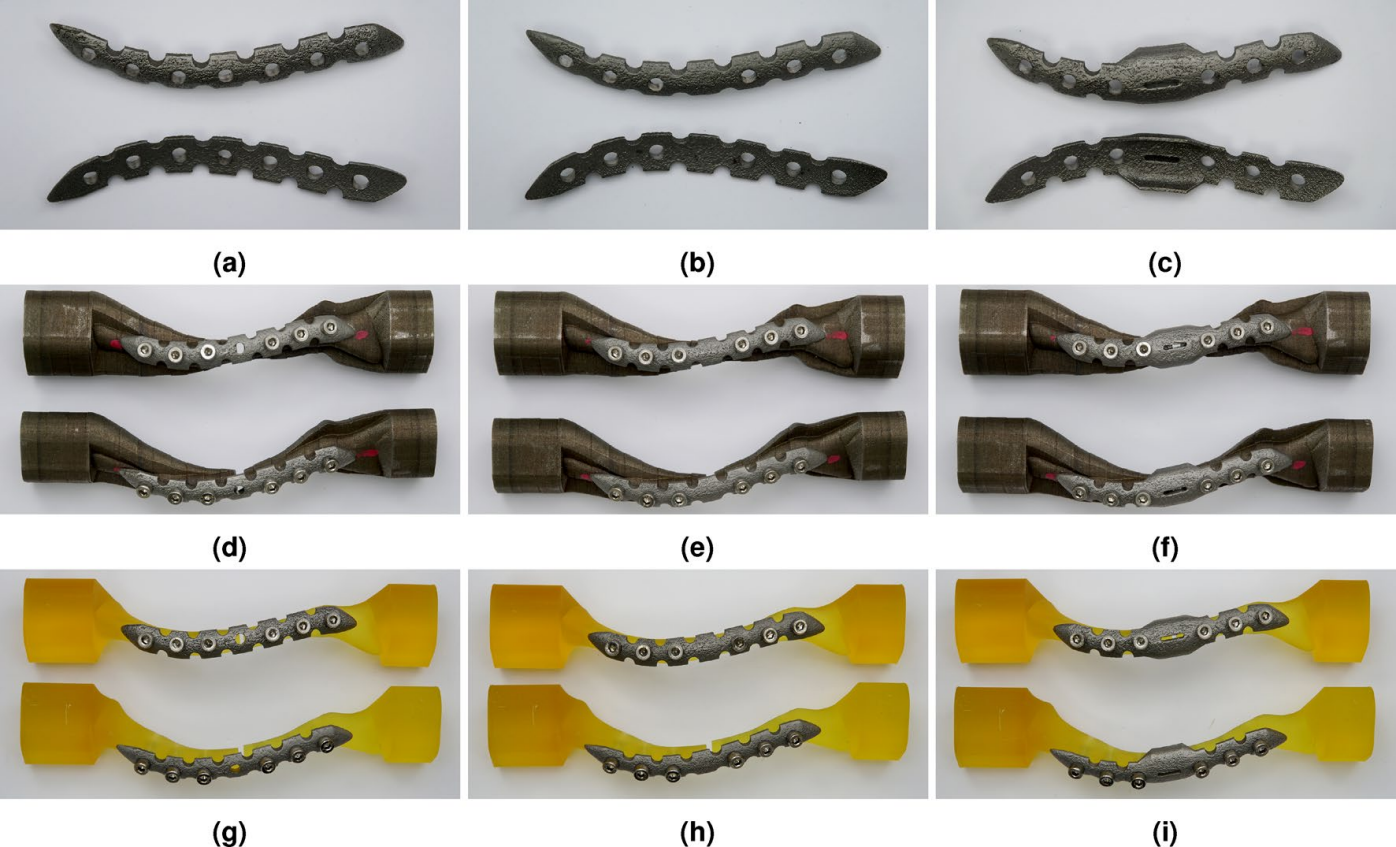
LCP and clavicle products made of metal and resin materials fabricated by 3D printers, (**a**) typical shape customized LCP (Type 1), (**b**) berried-hole shape customized LCP (Type 2), (**c**) double-curved wing shape customized LCP (Type 3), (**d**) Type 1 LCP with reinforced metal clavicle structure jig, (**e**) Type 2 LCP with reinforced metal clavicle structure jig, (**f**) Type 3 LCP with reinforced metal clavicle structure jig, (**g**) Type 1 LCP with bare resin clavicle structure jig, (**h**) Type 1 LCP with bare resin clavicle structure jig, (**i**) Type 1 LCP with bare resin clavicle structure jig.

On the biaxial testing machine (INSTRON 5982, USA), we conducted bending and compression tests at room temperature. These tests were performed after securely fastening the LCP and clavicle, with screws tightened using a digital torque wrench at a torque of 2.5 to 3.5 N$\cdot$m (Fig. 6). For the bending test, one end of the horizontal specimen was firmly fixed, and a single cantilever bending test was conducted using a cylindrical movable actuator with a diameter of 20 mm. As shown in Fig. 6a, the movable actuator was not affixed to the specimen, and bending was applied in the z-axis direction by applying a load from top to bottom. The displacement loading rate was set to 1mm/min. The LCP held the specimen facing upward, and the test was stopped once the upper and lower clavicles became attached due to bending. In the compression test, a cylindrical movable actuator with a diameter of 100 mm was used after securely fixing the bottom of the longitudinal specimen (Fig. 6b). The displacement loading mode was employed, and the loading rate was set at 1 mm/min. Similar to the bending test, the test was halted as soon as the top and bottom clavicles were attached.

We performed torsion testing using a BIAXIAL SERVOHYDRAULIC FATIGUE TESTING SYSTEM (INSTRON 8874). The torsion test was conducted with rotary displacement conditions as the control mode, and a ramp rate of 0.5 deg/s. The rotary torque limit of the torsion test device was set to 95 N$\cdot$m, with a rotation angle range of up to 120$^{\circ }$. The torsion test was carried out under the same conditions regardless of the clavicle material. As can be seen in Fig. 6c, a special device was manufactured to perform a torsion test by securely fixing the hold part in a jig with a reinforced clavicle structure.

**Figure 7:**
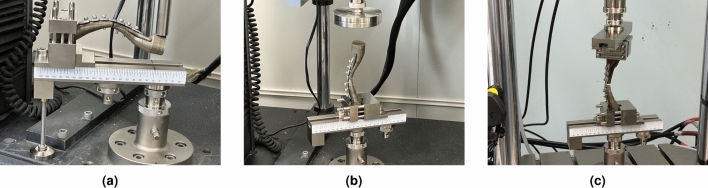
Further verification testing of the reinforced clavicle jig using a specially designed mechanical load test device, (**a**) cantilever bending test, (**b**) axial compression test, (**c**) torsion test.

## Results

The Fig. 7 shows the results of the FEA simulation, which analyzed the mechanical properties, displaying the distribution of equivalent stress. Under the cantilever bending force boundary condition, as the shape of the LCP transformed from type 1 to type 3, the size and range of stress concentration at the FZ point decreased. This decrease occurred rapidly when the LCP shape partially changed from type 1 to type 2. In the case of type 3, the point of stress concentration shifted from the FZ point to the M$_{3}$ and L$_{1}$ points when the cantilever bending force was applied. The stresses concentrated at the M$_{3}$ and L$_{1}$ points were lower than those of the typical structure. When an axial compression force was present, the shape changes in the LCP resulted in a decrease in stress concentration at the FZ point, as well as a reduction in the range of peak stress. Although the directions and forces differed, the force transmitted through the reinforced clavicle structure induced both bending and compression of the LCP simultaneously. As a result, stress concentration was alleviated similarly to the cantilever bending force under axial compression. However, the changes in stress concentration value at the FZ point under torsion torque conditions were relatively small, and irregular peak stress changes were also observed.

**Figure 8:**
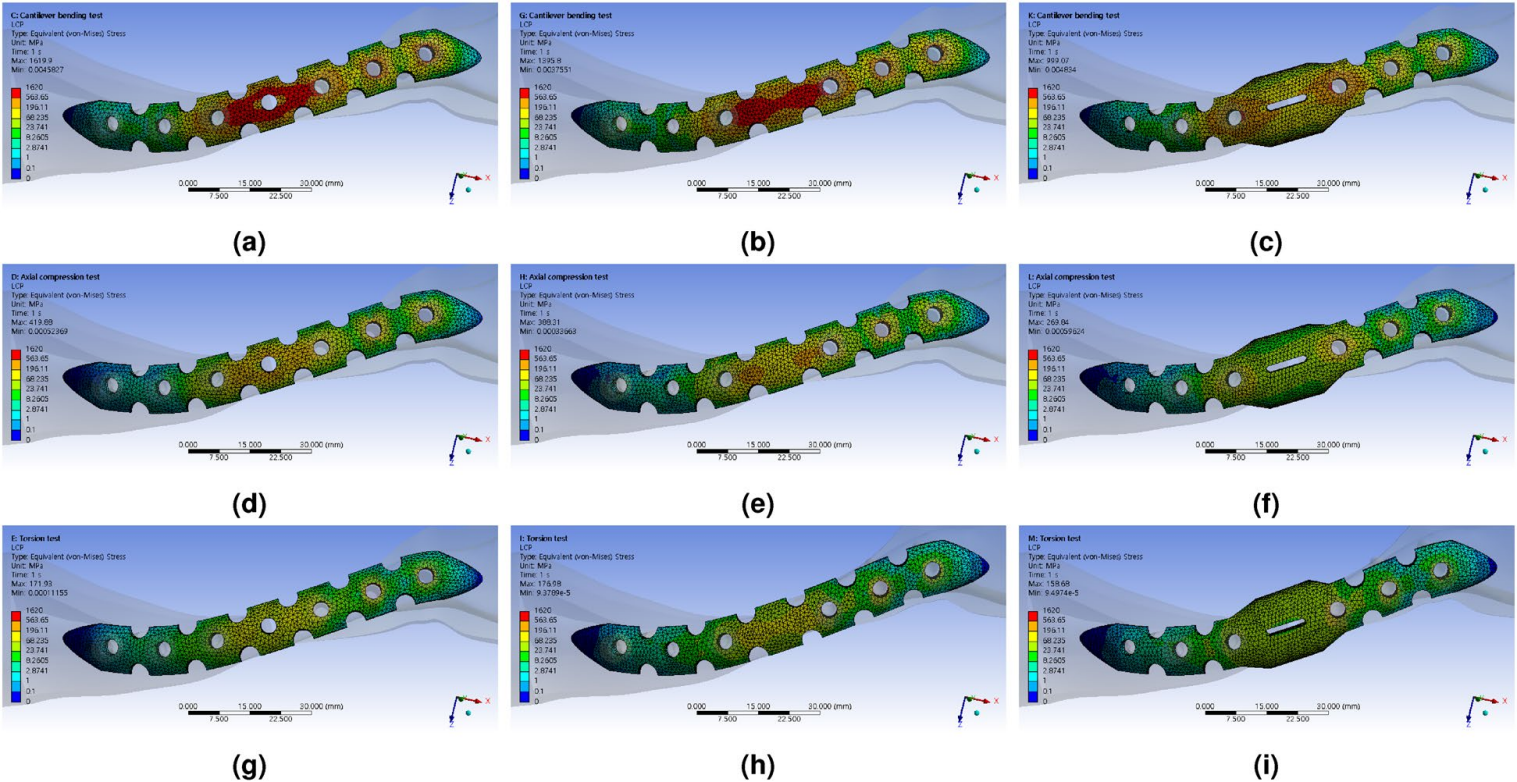
FEA simulation analysis results of the response to externally applied force to the LCP using the reinforced clavicle structure jig, Equivalent (von-Mises) Stress, (**a**) typical shaped LCP (Type 1) with cantilever bending force, (**b**) berried-hole shaped LCP (Type 2) with cantilever bending force, (**c**) double-curved partial wing shaped LCP (Type 3) with axial compression force, (**d**) typical shaped LCP (Type 1) with axial compression force, (**e**) berried-hole shaped LCP (Type 2) with axial compression force, (**f**) double-curved partial wing shaped LCP (Type 3) with axial compression force, (d) typical shaped LCP (Type 1) with torsion torque, (e) berried-hole shaped LCP (Type 2) with torsion torque, (f) double-curved partial wing shaped LCP (Type 3) with torsion torque.

**Table 4:** FEA simulation results according to hole location of customized LCP using a jig with a reinforced metal clavicle structure that responds to applied external force.

Tests	Cantilever bending			Axial compression			Torsion		
Points	1	2	3	1	2	3	1	2	3
$\sigma _{m}$M$_{1}^{1}$	3.756	3.475	4.178	1.140	1.033	1.138	1.640	1.568	1.703
$\sigma _{m}$M$_{2}$	19.798	19.305	20.871	6.802	6.501	6.802	10.449	10.566	10.692
$\sigma _{m}$M$_{3}$	288.392	349.440	333.539	71.841	85.274	81.941	40.009	50.947	54.154
$\sigma _{m}$FZ$^{2}$	1421.899	547.349	176.053	380.020	148.839	43.602	73.029	67.613	15.093
$\sigma _{m}$L$_{1}^{3}$	625.554	667.298	256.989	166.662	177.694	69.996	60.628	73.277	51.556
$\sigma _{m}$L$_{2}$	1082.767	632.162	240.363	290.985	169.944	61.748	55.592	44.075	33.399
$\sigma _{m}$L$_{3}$	833.584	842.027	323.422	224.038	226.292	82.959	39.538	37.780	26.524
$\sigma _{m}$ $\overline{M}^{4}$	103.982	124.073	119.529	26.594	30.936	29.960	17.366	21.027	22.183
$\sigma _{m}$ $\overline{L}^{5}$	847.301	713.829	273.592	227.228	191.310	71.567	51.919	51.711	37.160
$\sigma _{m}$ $\overline{L} / \sigma _{m}\overline{M}^{ 6}$	8.149	5.753	2.289	8.544	6.184	2.389	2.990	2.459	1.675
${{\sigma _{m}}^{{Full}^{7}}}$	2531.805	1395.759	999.070	698.577	388.309	269.836	196.271	176.978	158.680
${{\sigma _{m}}^{{LCP}^{8}}}$	1619.885	1395.759	999.070	419.883	388.309	269.836	171.929	176.978	158.680
$\varepsilon _{m}$M$_{1}^{1}$	0.031	0.030	0.036	0.009	0.009	0.010	0.014	0.013	0.014
$\varepsilon _{m}$M$_{2}$	0.171	0.153	0.167	0.057	0.052	0.054	0.086	0.083	0.085
$\varepsilon _{m}$M$_{3}$	2.317	2.819	2.771	0.576	0.689	0.680	0.330	0.407	0.444
$\varepsilon _{m}$FZ$^{2}$	11.568	4.211	1.354	3.091	1.145	0.335	0.595	0.520	0.116
$\varepsilon _{m}$L$_{1}^{3}$	5.463	5.726	2.214	1.453	1.524	0.607	0.511	0.606	0.442
$\varepsilon _{m}$L$_{2}$	8.398	5.323	2.021	2.257	1.430	0.519	0.434	0.375	0.289
$\varepsilon _{m}$L$_{3}$	7.038	6.665	2.584	1.892	1.791	0.663	0.368	0.304	0.216
$\varepsilon _{m}$ $\overline{M}^{4}$	0.840	1.001	0.991	0.214	0.250	0.248	0.143	0.168	0.181
$\varepsilon _{m}$ $\overline{L}^{5}$	6.966	5.905	2.273	1.867	1.582	0.596	0.438	0.428	0.316
$\varepsilon _{m}$ $\overline{L} / \varepsilon _{m}\overline{M}^{ 6}$	8.296	5.899	2.293	8.719	6.334	2.403	3.054	2.552	1.742
${{\varepsilon _{m}}^{{Full}^{9}}}$	20.516	11.543	8.593	5.641	3.129	2.200	1.607	1.399	1.380
${{\varepsilon _{m}}^{{LCP}^{ 10}}}$	12.625	10.761	8.593	3.244	2.994	2.200	1.357	1.399	1.380

Medial screw hole positions.

Fracture zone screw hole position.

Lateral screw hole position.

Average value of medial screw hole positions.

Average value of lateral screw hole positions.

Ratio of average value of medial and lateral screw hole positions.

Full scale range maximum stress(MPa).

LCP scale range maximum stress(MPa).

Full scale range maximum strain rate(mm/mm) $\times$ 1e3.

LCP scale range maximum strain rate(mm/mm) $\times$ 1e3.

To facilitate intuitive comparison with the upcoming verification experiment, the variance value of equivalent strain was extracted (Fig. 8). As anticipated, the most significant deformation occurred consistently at the FZ location across all types. Under the cantilever bending force, the deformation in type 1 primarily took place in the L$_{3}$ direction based on the FZ location. In type 2, a similar occurrence of strain asymmetry was observed, but the size of the peak strain area at the FZ point slightly decreased. In type 3, there was a noticeable improvement in the strain bias phenomenon based on the FZ position. The strain rate in a wide area decreased rapidly at the FZ point, and the strain concentrated near the window for confirming the fracture site. As a result, the location where deformation mainly occurred shifted to M$_{3}$ and L$_{1}$, and the deformation rate increased around the window of the FZ region. A similar trend was observed under the axial compression condition. However, when torsion torque was applied, it exhibited a different pattern from the other two force conditions. Essentially, the area where the maximum strain occurred was similar for all LCP shapes.

**Figure 9:**
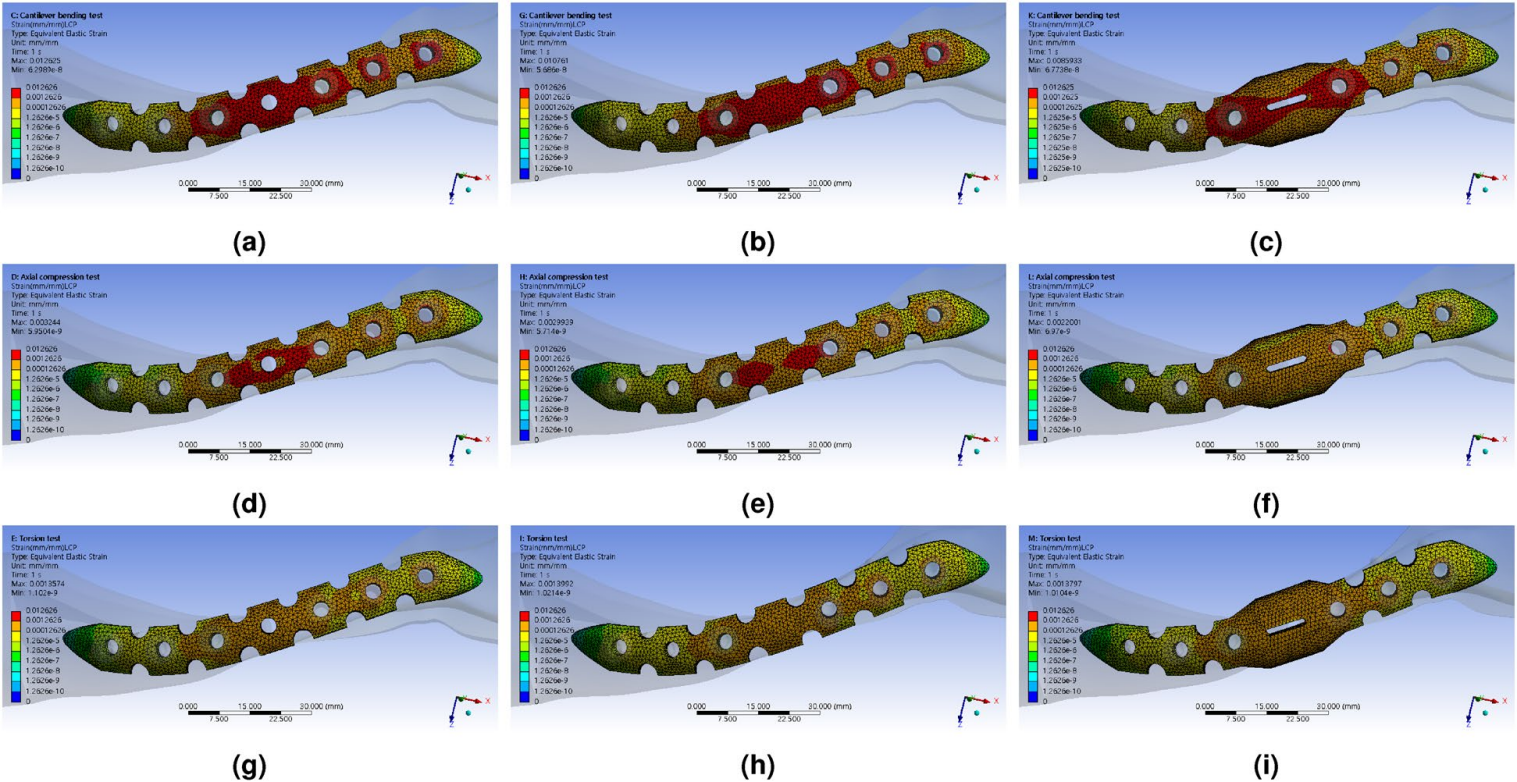
FEA simulation analysis results of the response to externally applied force to the LCP using the reinforced clavicle structure jig, Equivalent (von-Mises) Strain, (**a**) typical shaped LCP (Type 1) with cantilever bending force, (**b**) berried-hole shaped LCP (Type 2) with cantilever bending force, (**c**) double-curved partial wing shaped LCP (Type 3) with axial compression force, (**d**) typical shaped LCP (Type 1) with axial compression force, (**e**) berried-hole shaped LCP (Type 2) with axial compression force, (**f**) double-curved partial wing shaped LCP (Type 3) with axial compression force, (d) typical shaped LCP (Type 1) with torsion torque, (e) berried-hole shaped LCP (Type 2) with torsion torque, (f) double-curved partial wing shaped LCP (Type 3) with torsion torque.

**Figure 10:**
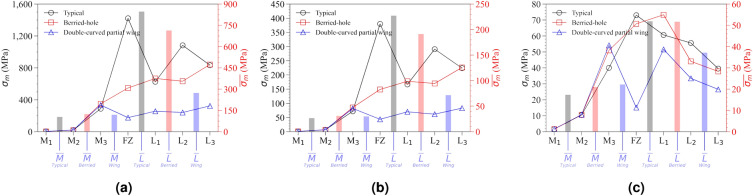
Distribution of $\sigma _{m}$ values by location of the LCP screw hole extracted from the mechanical property simulation results in response to externally applied force of the LCP using a reinforced clavicle jig, (**a**) cantilever bending force condition, (**b**) axial compression force condition, (**c**) torsion torque condition.

To quantitatively compare the magnitude of stress concentration according to the hole location of the LCP, stress and strain values were extracted and organized in Table 4. By comparing the stress distribution across the LCP, bone, and bolts, it becomes possible to easily identify which part experiences the highest stress concentration when examining the stress distribution of the LCP alone. To enhance ease of interpretation of the numerical data, fixed points were assigned based on the screw positions of the LCP, and the observed stress values were visualized (Fig. 9 and 10).

**Figure 11:**
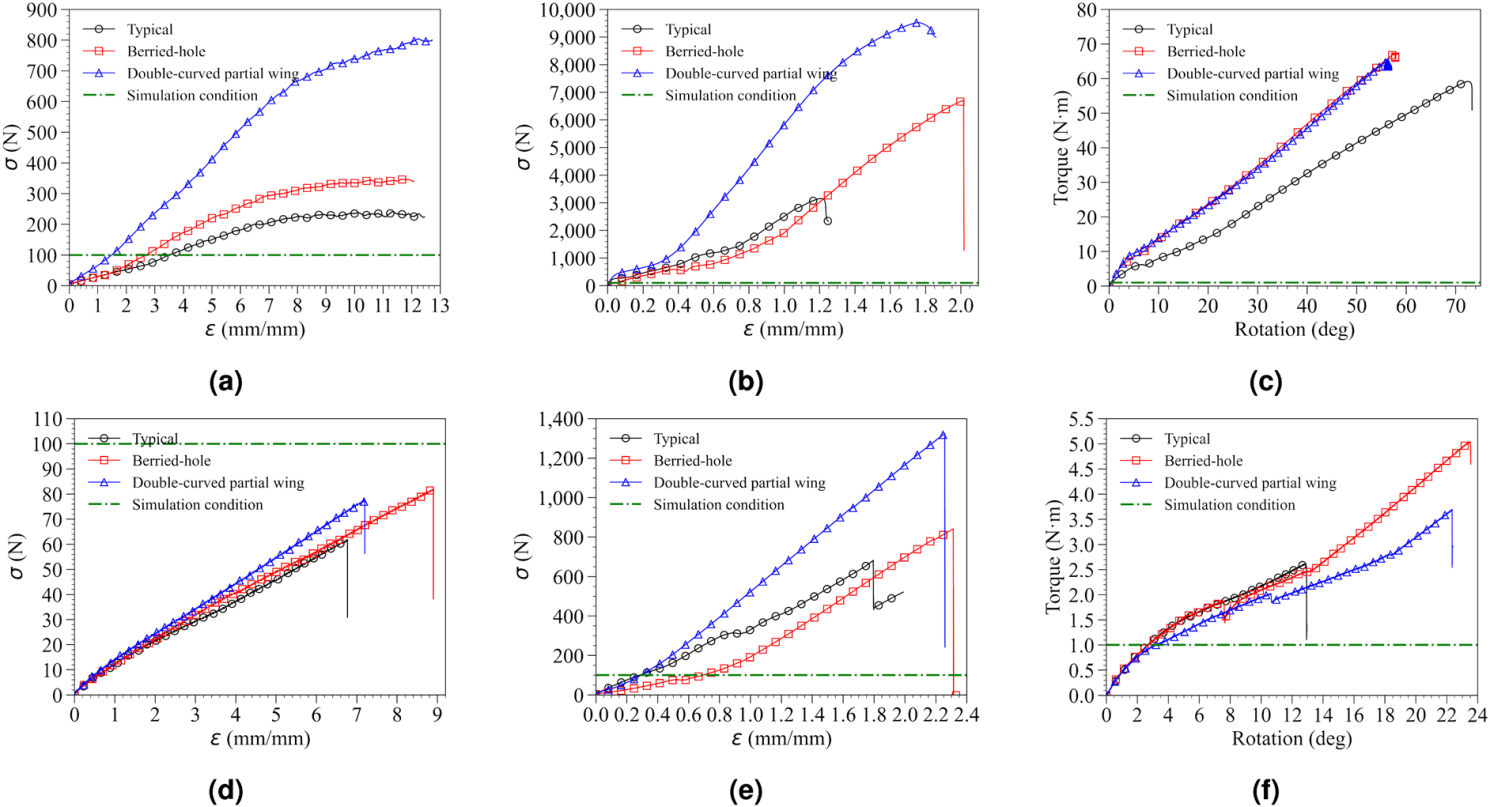
Stress-strain curve results of LCP coupled to the clavicle jig by applying a customized device for mechanical property testing using a universal material testing machine (UTM), (**a**) cantilever bending test result with reinforced metal clavicle jig, (**b**) axial compression test result with reinforced metal clavicle jig, (**c**) torsion test result with reinforced metal clavicle jig, (**d**) cantilever bending test result with bare resin clavicle jig, (**e**) axial compression test result with bare resin clavicle jig, (**f**) torsion test result with bare resin clavicle jig.

In order to quantitatively compare the mechanical properties based on the shape of the LCP, stress-strain curves of the LCP were obtained under different external force conditions (Fig. 11). Ultimate stress values were extracted for various LCPs subjected to each external force condition and summarized in Table 5.

**Figure 12:**
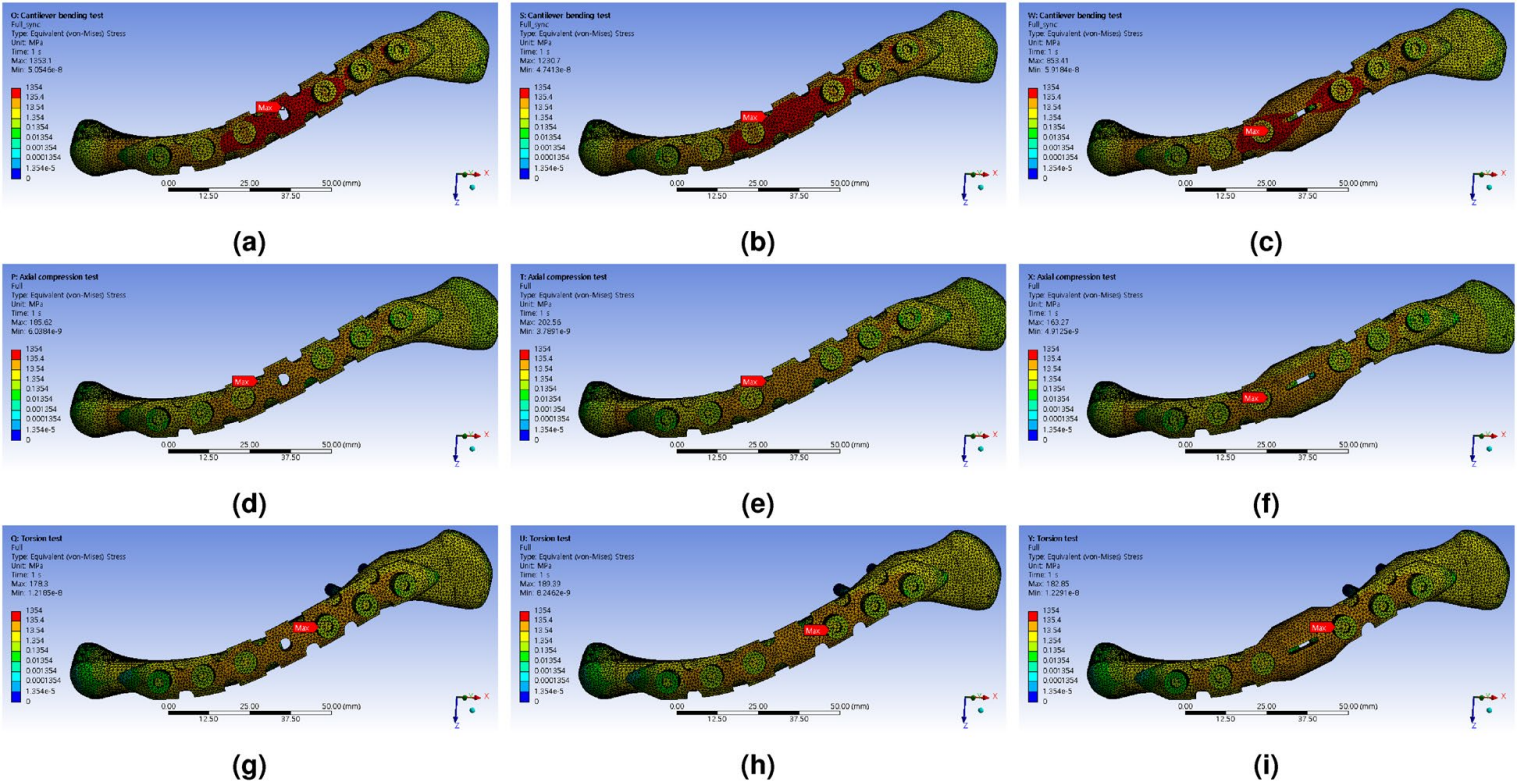
Distribution results of Equivalent (von-Mises) Stress for all parts extracted by performing FEA simulation analysis according to external force conditions of the natural clavicle bone structure model, (**a**) cantilever bending force with typical LCP shapes, (**b**) axial compression force with typical LCP shapes, (**c**) torsion torque with typical LCP shapes, (**d**) cantilever bending force with berried-hole LCP shapes, (**e**) axial compression force with berried-hole LCP shapes, (**f**) torsion torque with berried-hole LCP shapes, (**g**) cantilever bending force with double-curved partial wing LCP shapes, (**h**) axial compression force with double-curved partial wing LCP shapes, (**i**) torsion torque with double-curved partial wing LCP shapes.

**Figure 13:**
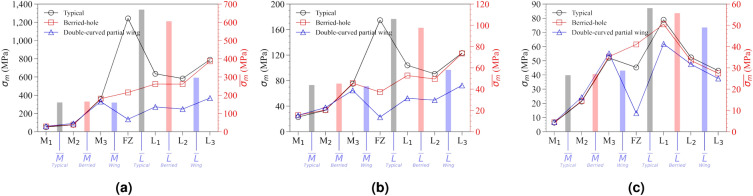
Distribution of $\sigma _{m}$ values by location of the LCP screw hole extracted from the mechanical property simulation results in response to externally applied force of the LCP using a natural clavicle bone structure, (**a**) cantilever bending condition, (**b**) axial compression condition, (**c**) torsion condition.

**Figure 14:**
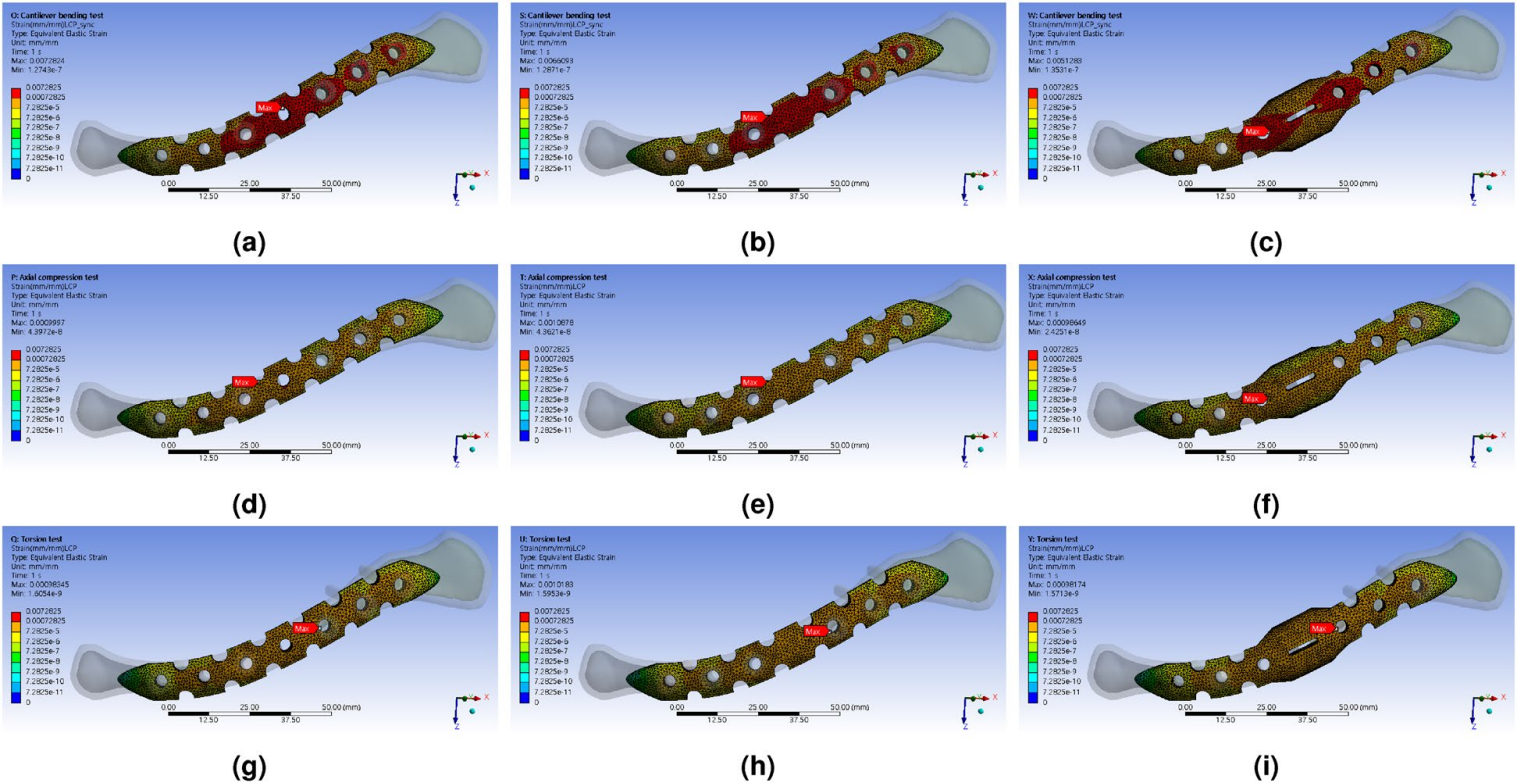
Distribution results for LCP parts of Equivalent (von-Mises) Strain extracted by performing FEA simulation analysis according to external force conditions of the natural clavicle bone structure model, (**a**) cantilever bending force with typical LCP shapes, (**b**) axial compression force with typical LCP shapes, (**c**) torsion torque with typical LCP shapes, (**d**) cantilever bending force with berried-hole LCP shapes, (**e**) axial compression force with berried-hole LCP shapes, (**f**) torsion torque with berried-hole LCP shapes, (**g**) cantilever bending force with double-curved partial wing LCP shapes, (**h**) axial compression force with double-curved partial wing LCP shapes, (**i**) torsion torque with double-curved partial wing LCP shapes.

The distribution of maximum stress ($\sigma _{m}$), calculated for the FEA simulation results of the structure applying actual clavicle properties, is shown in Figs. 12 and 13. It was confirmed that, similar to the reinforced clavicle structure, the strain at the FZ point tends to decrease as the shape of the LCP changes. Furthermore, in the double-curved partial wing structure, it was observed that the strain rate at the FZ point decreased rapidly, and the location of maximum deformation shifted in relation to the deformation of the LCP (Fig. 14).

**Table 5:** Tensile strength measurement results when external force is applied by combining the clavicle structure jig and LCP with a specially manufactured device through further verification tests.

Clavicle material	Validation test	Locking Clavicle Plate		
		Typical	Berried	Wing
Metal	Cantilever bending (N)	241.326	346.857	804.055
	Axial compression (N)	3155.857	6696.932	9522.986
	Torsion (N$\cdot$m)	59.212	67.009	64.975
Resin	Cantilever bending (N)	61.782	81.886	77.473
	Axial compression (N)	682.007	841.086	1324.532
	Torsion (N$\cdot$m)	2.612	5.041	3.701

**Table 6:** FEA simulation results according to hole location of customized LCP using a jig with a natural clavicle bone structure that responds to applied external force.

Tests	Cantilever bending			Axial compression			Torsion		
Points	Typical	Berried-hole	Wing	Typical	Berried-hole	Wing	Typical	Berried-hole	Wing
$\sigma _{m}$M$_{1}^{1}$	50.332	57.081	59.154	22.972	25.917	25.636	6.335	6.665	6.779
$\sigma _{m}$M$_{2}$	75.475	77.345	90.209	33.531	33.875	37.781	21.280	21.672	24.249
$\sigma _{m}$M$_{3}$	355.406	362.008	329.821	75.049	76.000	64.752	52.019	52.893	55.203
$\sigma _{m}$FZ$^{2}$	1242.431	430.127	135.839	174.776	62.016	22.774	45.283	61.520	13.089
$\sigma _{m}$L$_{1}^{3}$	634.777	522.650	272.165	103.999	87.947	52.364	78.891	75.789	61.924
$\sigma _{m}$L$_{2}$	583.436	521.520	247.637	90.587	82.515	49.463	52.406	50.183	47.585
$\sigma _{m}$L$_{3}$	789.708	773.144	368.827	123.209	122.632	72.537	43.016	41.156	37.493
$\sigma _{m}$ $\overline{M}^{4}$	160.404	165.478	159.728	43.851	45.264	42.723	26.545	27.077	28.744
$\sigma _{m}$ $\overline{L}^{5}$	669.307	605.772	296.209	105.932	97.698	58.121	58.105	55.710	49.001
$\sigma _{m}$ $\overline{L} / \sigma _{m}\overline{M}^{ 6}$	4.173	3.661	1.854	2.416	2.158	1.360	2.189	2.057	1.705
${{\sigma _{m}}^{{Full}^{ 7}}}$	1353.107	1230.718	853.413	185.621	202.564	163.272	178.298	189.385	182.846
${{\sigma _{m}}^{{LCP}^{ 8}}}$	1353.107	1230.718	853.413	185.621	202.564	163.272	178.298	189.385	182.846
$\varepsilon _{m}$M$_{1}^{1}$	0.297	0.334	0.347	0.136	0.152	0.151	0.039	0.040	0.041
$\varepsilon _{m}$M$_{2}$	0.430	0.427	0.499	0.191	0.187	0.209	0.118	0.120	0.133
$\varepsilon _{m}$M$_{3}$	1.992	2.029	1.894	0.425	0.430	0.377	0.295	0.299	0.311
$\varepsilon _{m}$FZ$^{2}$	6.710	2.308	0.729	0.944	0.333	0.122	0.253	0.330	0.070
$\varepsilon _{m}$L$_{1}^{3}$	3.804	3.182	1.612	0.626	0.537	0.304	0.459	0.442	0.382
$\varepsilon _{m}$L$_{2}$	3.442	3.080	1.465	0.537	0.490	0.294	0.320	0.306	0.289
$\varepsilon _{m}$L$_{3}$	4.373	4.228	2.048	0.682	0.671	0.403	0.240	0.231	0.210
$\varepsilon _{m}$ $\overline{M}^{4}$	0.906	0.930	0.914	0.251	0.256	0.245	0.150	0.153	0.162
$\varepsilon _{m}$ $\overline{L}^{5}$	3.873	3.497	1.709	0.615	0.566	0.333	0.340	0.326	0.294
$\varepsilon _{m}$ $\overline{L} / \varepsilon _{m}\overline{M}^{ 6}$	4.273	3.760	1.870	2.453	2.206	1.359	2.259	2.131	1.816
${{\varepsilon _{m}}^{{Full}^{ 9}}}$	13.466	12.076	6.773	2.863	2.874	2.915	1.345	1.345	1.381
${{\varepsilon _{m}}^{{LCP}^{ 10}}}$	7.282	6.609	5.128	1.000	1.088	0.986	0.983	1.018	0.982

Medial screw hole positions.

Fracture zone screw hole position.

Lateral screw hole position.

Average value of medial screw hole positions.

Average value of lateral screw hole positions.

Ratio of Average value of medial and lateral screw hole positions.

Full scale range maximum stress (MPa).

LCP scale range maximum stress (MPa).

Full scale range maximum strain rate (mm/mm) $\times$ 1e3

LCP scale range maximum strain rate (mm/mm) $\times$ 1e3.

## Discussion

The decrease in peak stress area occurred rapidly at the FZ point as the LCP’s shape partially changed from type 1 to type 2. It is believed that filling the screw hole at the FZ point helped alleviate the stress concentration transmitted from the cantilever bending force in the LCP and redirected it towards the medial direction. This relaxation of stress concentration observed most prominently at the FZ point. However, it was observed in type 3 that the stress concentration shifted from the FZ point to the M$_{3}$ and L$_{1}$ points when the cantilever bending force was applied. Nevertheless, due to the presence of the double-curved partial wing structure, the stresses concentrated at the M$_{3}$ and L$_{1}$ points were lower than those in the type 1 structure. Under the axial compression force, stress concentration was alleviated in a similar manner to the cantilever bending force.

The change in stress concentration at the FZ point under torsion torque conditions was relatively small, and irregular peak stress changes were also observed. It was observed that the change in type 2 resulted in the reinforcement of the surrounding area of the thin FZ hole, where stress concentrated during the twisting process, leading to a stress dispersion effect. Both type 1 and type 2 structures had a basic 2D surface shape and showed high resistance to deformation in only one axis. As a result, the stress concentration pattern throughout the entire LCP area was similar, except for the alleviation of stress concentration at the FZ location. The type 3 structure, on the other hand, exhibited strong resistance to deformation in two or more axial directions, which effectively reduced the occurrence of stress concentration phenomena.

As predicted by the simulation results of the peak stress distribution, the FZ location experienced the most deformation under the cantilever bending condition in all shapes. Notably, in type 3, the strain bias phenomenon improved significantly based on the FZ position. When subjected to axial compression conditions, the actual deformation results displayed a significant difference across the entire LCP area, consistent with the findings of our previous study^9^. These results effectively address vulnerabilities that may arise at the FZ point in typical LCP structures and reduce the strain rate in the normal direction.

When torsion torque was applied, a different pattern was observed compared to other external force conditions. Due to the strain rate in the twisting process having the same legend range, it was challenging to visually compare the sizes accurately. To examine the strain distribution during torsion torque, Fig. 8g–i demonstrate a distinct pattern compared to other external force conditions. Generally, it can be observed that the area with the highest strain is similar across all LCP shapes. However, due to the strain rate in Fig. 8 having the same legend range, visually comparing sizes accurately is difficult.

The distribution results of stress and strain in a 3D shape provide the advantage of intuitively understanding the locations where stress and strain concentrate and dissipate under different external force conditions. However, it can be challenging to discern clear differences. To overcome this, we fixed the same position in the global coordinate space alongside the LCP screw hole and observed the stress magnitude. We defined observation points at three locations on each side of the fracture point and observed the stress values at the same location regardless of changes in the LCP shape. In type 3, an observation point was assigned to the window’s edge at the FZ location to compare the phenomena arising from the window shape.

Under the cantilever bending force, maximum stress ($\sigma _{m}$) decreased significantly from 1421.899 MPa to 176.053 MPa, as the LCP shape changed from type 1 to type 3. This decrease in stress is calculated to result in a stress dispersion effect nearly ten times stronger. Moreover, the average stress value in the medial direction is lower than that in the lateral direction. This is due to the medial direction joint acting as a fixed support and being the furthest point from the applied external force. In the case of type 1 under cantilever bending force conditions, it was observed that the stress concentration ratio differed by 8.149 times in both directions. However, this stress imbalance is reduced by 2.289 times in type 3, resulting in a stress imbalance relief effect of 356% compared to type 1.

In addition, by comparing the stress analysis results for all structures, including the clavicle, bolt, and LCP, with the stress analysis results for only the LCP, it is possible to determine which part the stress is concentrated in due to the applied external force. If we obtain the values of $\sigma _{m}^{Full}$ and $\sigma _{m}^{LCP}$, we could calculate the difference between them. While the maximum amount of stress transmitted to the LCP was 1619.885 MPa, the maximum value of the total stress in the FEA simulation was 2531.805 MPa. This indicates that the maximum stress was concentrated on the clavicle in type 1. If the maximum stress is concentrated on the clavicle, it may lead to additional fractures and cause the fixed LCP to fall off. Therefore, it is recommended to avoid such situations.

When type 2 was applied, it can be observed that the values of $\sigma _{m}^{Full}$ and $\sigma _{m}^{LCP}$ are the same. This means that the maximum stress concentration was located in the LCP as a result of the FEA simulation analysis. Due to the change in the structure of the LCP, the point of maximum stress concentration could be altered. Furthermore, the magnitude of the maximum stress concentrated in the LCP was found to be further reduced in type 3. Through the FEA simulation analysis, we extracted and organized the maximum strain values for the entire system and only the LCP parts, denoted as $\varepsilon _{m}^{Full}$ and $\varepsilon _{m}^{LCP}$, respectively.

By comparing the values of $\varepsilon _{m}^{Full}$ and $\varepsilon _{m}^{LCP}$, we can analyze the location where the greatest deformation occurs. Specifically, under cantilever bending force conditions in the type 3 structure, it is apparent that the maximum strain rate occurs exclusively within the LCP structure. This differs from the case of maximum equivalent stress. Since strain rate reflects the mechanical properties of the material and calculates the response to applied stress, it provides a more accurate means of identifying areas prone to deformation.

The results of the FEA simulation for axial compression force were similar to those for the cantilever bending force. This similarity is believed to be due to the consistent direction of the externally applied force in both cases. However, when torsion torque is applied, the results differ from other external force conditions. In this case, the stress concentration phenomenon at the FZ point is alleviated, albeit to a relatively small extent, resulting in the displacement of the location of the maximum stress point. Overall, the points of maximum stress and strain were calculated to be located within the LCP structure, with the exception of type 1.

In type 1 structure, the highest stress was concentrated at the FZ location. The stress concentration at the FZ point rapidly decreased as type 2 plate was applied, and the stress at the L$_{2}$ point also decreased. Finally, when the type 3 plate was applied, the stress concentration effect in the lateral direction was greatly reduced, and this was believed to be because the externally applied load was effectively distributed through the LCP and transmitted in the medial direction. As a result, the maximum stress concentration moved to the M$_{3}$ position, and the maximum stress value at this time also shows the lowest value. As a result, it would be easier to preserve the fixation of surgery using type 3 plate compared to type 1 plate under cantilever banding force. When axial compression force was used, similar pattern to the results of cantilever bending was found. However, pattern of change occurred in a very complex manner where torsion torque was applied. When type 2 plate was applied, it was confirmed that the point of maximum stress generation moved from the FZ to the L$_{1}$, and the stress was transferred in the medial direction, so that the stress distribution in both directions was more evenly distributed. When using the type 3 plate, the magnitude of the maximum stress decreased rapidly at the FZ location, but the maximum stress point moved to the M$_{3}$ and L$_{1}$ positions. The reason why the stress concentration effect rapidly decreased at the FZ point was because type 3 structure had strong resistance to bending in more than two axial directions and thus was able to well resist the direction of the applied force.

When cantilever bending force was applied, the difference in mechanical properties according to each LCP shape was dramatically different. Assuming that the same external force as the boundary condition of the FEA simulation was applied, the LCP of type 1 plate showed the highest strain rate at 3.493 mm/mm, and type 2 shape showed a slightly lower value of 2.680 mm/mm. Type 3 structure showed a strain rate of 1.483 mm/mm, which was 2.36 times less than type 1 plate. This tendency was confirmed more clearly when comparing the mechanical properties of the LCP shape itself after the elastic limit region, unlike the boundary conditions of FEA simulation.

According to the test using a reinforced metal clavicle jig, ultimate stress increased 3.33 times from 241.322 N to 804.057 N as the LCP changed from type 1 to type 3. The experimental results for cantilever bending force condition had similar tendencies when compared to the $\sigma _{m}^{Full}$ results. The value of $\sigma _{m}^{Full}$ generated from type 1 plate was 2531.805 MPa, and type 3 plate was 990.070 MPa, showing a stress reduction effect of 2.55 times. Although a slightly smaller percentage of stress relaxation effect than the calculated results was actually observed, this was judged to be an excellent FEA simulation prediction model, considering cases that deviate from ideal boundary conditions that might occur in the actual experimental process. When performing FEA simulations using a verified model that considers the characteristics of the actual patient’s clavicle and surgical material, mechanical properties that may occur in a clinical trial environment can be more accurately predicted.

Mechanical properties were measured under compression conditions based on the long axis of the clavicle. The measured results showed a complex aspect, unlike the cantilever bending force, which was because the two forces of compression and bending act in a complex manner due to the geometry of the combination of the fractured clavicle jig and the LCP under compression conditions. Therefore, in order to interpret the experimental results, mechanical properties could be compared based on the ultimate stress point where large deformation occurred. The lowest ultimate stress was observed at 3,155.858 N in type 1 plate. The highest ultimate stress of 971.074 MPa was confirmed in type 3 structure, which was observed to have resolved the stress concentration phenomenon by 3.02 times compared to type 1. The value of $\sigma _{m}^{Full}$ was calculated as 698.577 MPa for type 1 and 269.836 MPa for type 3, showing a 2.59 times stress concentration improvement effect. In the axial compression test, a slightly higher stress concentration improvement effect occurred compared to the calculated results, but this was believed to be an error caused by not reflecting the nonlinearity of the material that actually occurred because the FEA simulation was calculated before the elastic limit. However, the reliability of the FEA simulation is accurate even though these errors.

In the case of the torsion test, a different aspect was seen from the previous two conditions. Because the stiffness of the reinforced metal clavicle was high, the change in torque value due to angle change occurred rapidly at the beginning, making it difficult to compare simulation conditions. Stress was concentrated on the fixed stainless-steel bolt and failure occurred on the principle of a lever, making it difficult to confirm the destruction of the LCP in the current system. In conclusion, linear responsiveness was confirmed within the elastic limit of LCP. The results of the FEA simulation showed that the value of $\sigma _{m}^{Full}$ was 196.271 MPa in type 1 and 158.680 MPa in type 3, and an improvement of 1.23 times occurred. The ultimate stress value of the torsion test was measured to be 580.671 N in type 1 and 637.187 N in type 3. This means that the stress concentration phenomenon of 1.10 times had been alleviated in the LCP of type 3 plate and showed similar values to the calculation results of the simulation.

When checking the results of comparing $\sigma _{m}^{Full}$ and $\sigma _{m}^{LCP}$, the part of the maximum stress was located in LCP in all cases. However, the size of the strain rate was calculated differently in all cases by comparing the values of $\varepsilon _{m}^{Full}$ and $\varepsilon _{m}^{LCP}$. The reason why the value of $\varepsilon _{m}^{LCP}$ was smaller overall might be that the stiffness of titanium alloy is 10.97 times greater than that of cortical bone and 186.4 times greater than that of cancellous bone.

Unlike the simulation results of the reinforced metal clavicle, it was confirmed that the high stress concentration effect occurring at the L$_{2}$ position was not observed when the actual clavicle properties were applied. It can be seen that most of the stress is concentrated in the lateral direction in the typical LCP under the conditions of cantilever bending force and axial compression, and a tendency to decrease as it changes to type 3 shape is consistent with previous simulations. In contrast, in the torsion torque condition, the difference is that the point of maximum stress generated in the typical LCP has moved from FZ to L$_{1}$.

When a resin clavicle was used, the mechanical properties were analyzed until failure occurred. In the cantilever bending test, failure occurred below 98.067 N in all cases, which was lower than the boundary condition value of the simulation. The reason why such damage occurred was because the resin material used had a high resistance to compressive force, but cracks easily occur in the case of tensile force due to the brittle nature of the material. Therefore, when analyzing the stress-strain curve using a resin clavicle, it was necessary to consider changes in the shape of the LCP by comparing the slope values rather than directly comparing the ultimate stress values. Because the destruction of the resin clavicle occurred faster than the deformation of the LCP, the results were obtained by comparing the properties within the elastic limit area of the LCP. Considering that the size of the ultimate stress at which the clavicle was broken was similar to the boundary condition value of the simulation, it was confirmed that the boundary condition of the simulation was within the range where additional fracture of the clavicle was actually possible. This tendency showed a similar pattern in the axial compression test, but the resin material had strong characteristics against compressive force, showing similar characteristics to a reinforced metal clavicle. Due to these characteristics, damage occurred when the boundary conditions set in the simulation were exceeded. In addition, the ultimate stress value was measured at 682.003 N in type 1 and 1324.525 N in type 3 shape, confirming the effect of relieving stress concentration by 1.94 times. Under torsion condition, the mechanical properties were almost the same at low rotation angles as the shape of the LCP changed. At angles above 14 degrees, failure occurred regardless of the shape of the LCP, but the difference in ultimate stress was very small compared to the reinforced metal clavicle. This result was similar to the simulation results.

**Figure 15:**
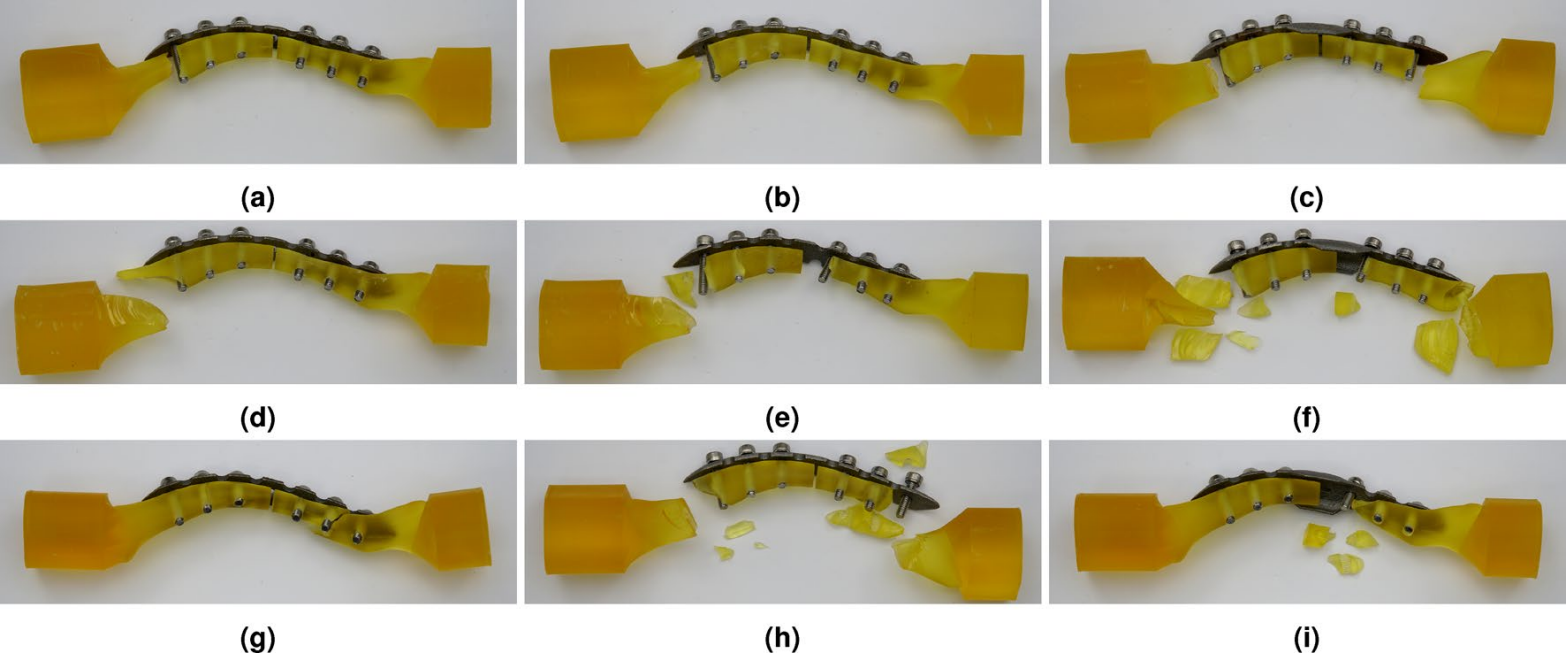
Fractured specimen shapes as a result of further verification test of customized LCP mounted on bare resin clavicle jig, (**a**) cantilever bending test with typical LCP shape, (**b**) cantilever bending test with berried-hole LCP shape, (**c**) cantilever bending test with double-curved partial wing LCP shape, (**a**) axial compression test with typical LCP shape, (**b**) axial compression test with berried-hole LCP shape, (**c**) axial comrpession test with double-curved partial wing LCP shape, (**a**) torsion test with typical LCP shape, (**b**) torsion bending test with berried-hole LCP shape, (**c**) torsion bending test with double-curved partial wing LCP shape.

As a result of validation using a resin bone, the shape and location of the breakage varied depending on the type of external force applied (Fig. 15). According to the shape of the broken clavicle, the form in which stress was concentrated in the M$_{1}$ area and the damage occurred can be confirmed under the cantilever bending and axial compression force because stress was concentrated in the medial direction from the FZ point. In contrast, when type 3 plate was applied, damage was distributed to two points. The results of stress being distributed in type 3 plate and concentrated at two or more points based on the FZ point were confirmed in Table 6, showing a similar trend to the validation test. The results of the torsion test showed that the destruction progressed in a complex form. In type 1 structure, a crack occurred at point M$_{1}$, and it was fractured simultaneously at points M$_{1}$ and L$_{3}$ in type 2. In type 3 structure, it was confirmed that stress was concentrated at the L$_{1}$ point and failure occurred. This irregularity of the failure point can be explained by the difference in the values of $\varepsilon _{m}^{Full}$ and $\varepsilon _{m}^{LCP}$.

There are some limitations. First, the fracture is complex and very complicated in the real world, and the actual number of fracture cases. Second, there are several considerations that could not be embodied in simulation. Some of the implementable difficulties include changes in clavicle anatomy, micro-motion between bones and plates, stress-raising effects of screws, and bone quality. To simplify the simulation, these considerations were excluded. The cancellous bone structure present in the actual clavicle was deleted and everything was designed and manufactured in the form of cortical bone. No matter how distinctly designed, and 3D printed, the actual bone cannot be fully implemented.

## Conclusions

Gradual passive and active-assisted range of motion can be initiated 1 week after surgery and should continue until 6 week after surgery. The patient then should begin more aggressive active range of motion and light lifting after postoperative 6 week according to the typical rehabilitation protocol^31^. The patient then should begin more aggressive active range of motion and light lifting after postoperative 6 week. This new LCP design reduces the stress concentration on the fracture site and amount of stress in the fracture area while applying cantilever bending force. Therefore, it is believed that the duration of rehabilitation protocols can be accelerated compared to when surgery was performed using a traditional LCP.

## Data Availability

The datasets used and/or analysed during the current study available from the corresponding author on reasonable request.
